# Lessons Learned from Implementing Culturally Sensitive Adaptations to a Nutrition Education Program in Four Anishinaabe Communities

**DOI:** 10.1016/j.cdnut.2024.104496

**Published:** 2024-10-29

**Authors:** Erin MB Tigue, Dawn S Earnesty, Ronald L Gibbs, Beth I Sieloff

**Affiliations:** 1 Michigan State University Extension, Health and Nutrition East Lansing, MI, United States; 2 Inter-Tribal Council of Michigan, Sault Saint Marie, Michigan, United States

**Keywords:** nutrition education, cooking class, Indigenous food, cultural adaptation, food sovereignty

## Abstract

**Background:**

The Anishinaabe ways of eating healthy and staying physically active are not well represented in federally funded health education programs such as Supplemental Nutrition Assistance Program Education (SNAP-Ed). The curricula approved for use by SNAP-Ed implementing agencies were not developed with Anishinaabe foods, cultures, and traditions in mind.

**Objectives:**

This paper shares the process and lessons learned from a collaborative effort to adapt recipes from a SNAP-Ed-approved nutrition and cooking education program, Cooking Matters at Home (CMAH), to reflect cultural preferences and traditions of participants from 4 Anishinaabe tribal communities in Michigan.

**Methods:**

University Extension instructors, Tribal health staff, and an intertribal organization codesigned and implemented the adaptation. Extension instructors facilitated 4 separate series of cooking and nutrition classes using CMAH curriculum with 4 Tribes. A descriptive, cross-sectional evaluation design focused on participant engagement and recipe adaptation. Evaluation included formative interviews with Tribal health staff, postintervention community participant focus groups to assess program satisfaction and cultural acceptability of adaptations, and process interviews with Extension instructors and Tribal health staff.

**Results:**

Between October 3 and December 7, 2022, classes reached 30 adults across 4 sites. The mean class size was 7–8 participants. Eighty-six percent of participants attended every class session offered in their community. Eight recipes were adapted to include traditional foods (e.g., wild rice and maple syrup); 11 were prepared as written. A challenge was access and availability of some traditional recipe ingredients. Participants expressed a range of food knowledge and behaviors that reflect diverse histories and cultures within each community. Tribal staff and participants suggested that Extension instructors build rapport with community members, approach activities and participants with curiosity rather than sharing "expert" advice, implement more continuous programming, and improve cultural sensitivity.

**Conclusions:**

SNAP-Ed programs should support access to local Indigenous foods and make space for both community and individual traditions.

## Introduction

In spring 2023, a group of Indigenous scholars and community members presented a report to the United Nations on the Indigenous Determinants of Health [1]. The report stated that “land, culture, and language are powerful tools for healing” (1, pg. 6) among Indigenous communities around the world. Although every Indigenous nation, community, and individual has unique cultural practices and history, the report highlighted how several cultural factors common to Indigenous peoples around the world can support positive health outcomes. Supporting access to and use of traditional foods is one example. Definitions of traditional foods vary, but Rocillo-Aquino et al. [2] described 4 common dimensions of traditional food, including a connection to place (e.g., local), time (e.g., intergenerational and seasonal), know-how (knowledge of how to prepare food passed through generations), and cultural meaning (having a symbolic value and place in celebrations, ceremony).

In Michigan, where the work described in this article took place, there are 12 federally recognized Tribes across the state consisting of Chippewa (Ojibwe), Ottawa (Odawa), and Potawatomi (Bodowadomi) nations, referred to as the Three Fires Confederacy or Anishinaabeg. Before European colonization of North America, Indigenous Anishinaabe diets consisted primarily of seasonal foods local to the area, such as wild rice, maple sugar, berries, venison, and fish, among others [3,4]. Foods were acquired through hunting, fishing, foraging, and gardening, but also through trading with other tribal communities. Knowledge of how to cultivate, acquire and prepare foods was shared across generations. After Europeans arrived, colonization, forced assimilation, removal from homelands, and loss of Indigenous food sources occurred over generations. This resulted in a loss of knowledge and access to foods traditionally eaten by Anishinaabe peoples and some of the negative health disparities seen today between American Indians and nonmarginalized populations [5,6].

Health interventions that strengthen Indigenous identities, elevate traditional languages, and reconnect people with land and Indigenous food systems could help to address health disparities between American Indian/Alaska Native (AI/AN) and nonmarginalized populations [7,8]. Addressing health disparities in AI/AN communities in culturally sensitive ways has become a growing focus of federally funded food assistance programs. The 2018 Farm Bill expanded federally recognized Tribes’ authority to purchase agricultural commodities that provide nutritious, traditional foods to income-eligible Tribal citizens through the Food Distribution Program on Indian Reservations (FDPIR) [9]. Likewise, the USDA Supplemental Nutrition Assistance Program Education (SNAP-Ed), which provides states with funding to teach low-income families and individuals how to cook and eat healthy foods, has directed state and local implementing agencies to work with federally recognized Tribes to implement culturally relevant programming [10,11]. Despite these efforts and the growing global recognition of the role of culture and traditional foods in supporting Indigenous health, very few federally funded health education programs implemented in AI/AN communities incorporate the language, traditional foods, or other cultural factors unique to each Tribal Nation and community in the United States. Using curriculum that is not culturally appropriate can result in programming that is not effective or well accepted by participants and fails to incorporate unique cultural factors that could support optimal health outcomes [12,13]. Among the 132 evidence-based SNAP-Ed interventions listed in the online SNAP-Ed Resource Library and approved for use by implementing agencies, only 3 indicate they were designed specifically to be culturally appropriate to AI/AN audiences [[14], [15], [16], [17], [18]]. No curriculum that was listed in the online database and approved for broad use by all SNAP-Ed implementing agencies was designed specifically with or for Anishinaabe communities in Michigan.

In Michigan, 2 SNAP-Ed implementing agencies, Public Health Authorities of the state’s federally recognized Tribes, 3 tribally affiliated land grant colleges, and intertribal organizations all provide nutrition education programs in tribal communities. One of the SNAP-Ed implementing agencies is the university cooperative extension service involved in this study. At the time of this study, the other SNAP-Ed implementing agency in the state was piloting a food and nutrition curriculum developed specifically by and for Anishinaabe communities that features traditional seasonal foods and cultural values. That curriculum was originally developed to be implemented by Indigenous educators with knowledge of Anishinaabe culture and foodways [19]. There was little documentation of the cultural appropriateness of other SNAP-Ed-approved curricula that were developed for general audiences and available to both Indigenous and non-Indigenous educators for implementation in Anishinaabe communities. The Cooking Matters suite of programs is one such curriculum that can be implemented by non-Indigenous Extension instructors in partnership with tribal staff in Anishinaabe communities in Michigan. The purpose of this paper was to share the process and lessons learned from one effort to adapt a Cooking Matters curriculum to include more recipes with foods traditional to Anishinaabe tribal communities.

Cooking Matters (CM) is an evidence-based, nationally recognized nutrition and cooking education program with several versions tailored to different audiences, such as adults, families, and teens [20]. Each curriculum in the CM suite provides participants with hands-on education about how to prepare healthy, family-friendly, low-cost meals. After each weekly lesson, participants receive a bag of groceries to take home and practice a new recipe. The standard CM curricula for adults and families requires 2 h of instruction and cooking demonstrations each week for 6 wk. The time commitment can be a barrier for some participants as well as facilitators. The newest CM curriculum, Cooking Matters at Home (CMAH), was designed to be more flexible than other CM curricula in both implementation logistics and lesson content. CMAH can be virtual if needed, have shorter weekly sessions, be more flexible in which lesson topics are selected, and requires less evaluation paperwork than other CM curricula. Michigan State University (MSU) Extension, one of the state’s 2 SNAP-Ed implementing agencies, had implemented CMAH with general SNAP-Ed audiences but had not tested it specifically with tribal communities in the state at the time of this study. Furthermore, the approved recipes provided for all CM programming were not designed to reflect Anishinaabe communities and foodways.

A collaborative effort in 2022 aimed to address these gaps in the following 2 ways: *1*) by evaluating the feasibility of implementing the CMAH curriculum with 4 Anishinaabe communities and *2*) adapting and piloting recipes from the CM online recipe database to include traditional Anishinaabe foods. Although participant behavioral outcomes were measured, the focus of the evaluation and results described in this article was on the community participant and Tribal health staff engagement and satisfaction with the program and participant perspectives on cultural acceptability of recipe adaptations. This article is written primarily from the perspective of non-Indigenous researchers at MSU Extension, which is a land grant university and one of the state’s 2 SNAP-Ed implementing agencies. The InterTribal Council of Michigan (ITCM) and Tribal health staff from 4 participating Tribal Nations provided additional input and article review.

## Methods

### Setting

This adaptation was developed and implemented through a collaboration between MSU Extension’s SNAP-Ed funded nutrition education staff (“Extension instructors”), an intertribal organization, and community health educators (“Tribal health staff”) at 1 Ojibwe, 1 Odawa, and 2 Potawatomi Tribal Nations across Michigan. The 4 Tribal health centers had previously participated in a produce prescription program and educational programming to improve healthy, traditional food access through FDPIR via a grant administered by ITCM. The Tribal health staff and ITCM were interested in complementing these efforts with a hands-on nutrition and cooking class for adults and families that use more traditional foods in recipes. ITCM reached out to MSU Extension on behalf of the 4 Tribes to explore a collaborative effort to implement such a program. Extension instructors funded by SNAP-Ed are approved to implement a variety of nutrition education curricula. The project team, including Extension and Tribal health staff and ITCM, chose to use CM curriculum because of its hands-on design and popularity with tribal members when implemented in the past. CMAH was specifically chosen for this intervention because of its relative flexibility and because it had not yet been piloted and evaluated for cultural relevance to Anishinaabe communities, as described previously in this article.

### Study design

A descriptive, cross-sectional study design was utilized with a focus on program adaptation. Three program evaluation researchers and one student intern from Extension conducted formative research and program evaluation. All were trained in quantitative and qualitative evaluation methods as well as human research ethics. Tribal health staff informed the design and adaptation of the intervention, whereas Extension instructors facilitated the weekly classes.

**FIGURE 1:**
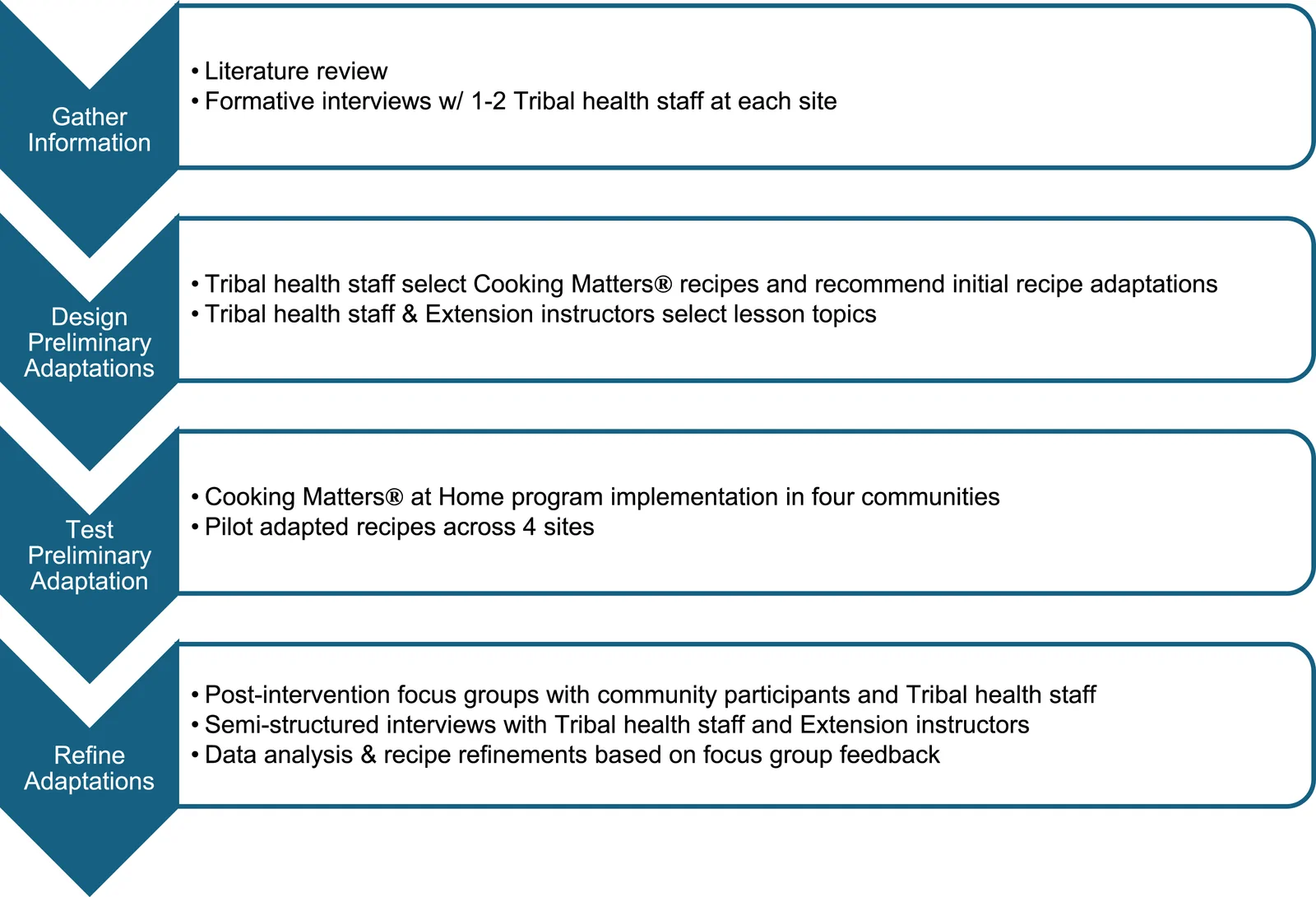
Program adaptation process.

Program adaptation was designed through an iterative process that aligns with the stages of cultural adaptation for evidence-based interventions as described in Barrera and Castro [12] and Barrera et al. [13]. Stages include information gathering, designing preliminary adaptations, preliminary adaptation, and refining adaptations (Figure 1).

#### Gather information

Information gathering occurred through a literature review and informal interviews with Tribal health staff. The evaluation team reviewed literature describing cultural adaptations to nutrition education programs generally and for Indigenous populations, as well as information on traditional foods and practices of Anishinaabe peoples. Informal formative interviews with 1 to 2 Tribal health staff at each site were implemented to collect information on challenges and successes with past CM programs in their community, desired outcomes for this program, and potential recipe adaptations. The formative interviews also informed the development of evaluation questions for postintervention focus groups and interviews, used for adaptation refinement. Formative interview responses primarily related to desired engagement and participant outcomes. Tribal health staff shared that they would like to improve participant retention compared with previous CM classes, maintain high engagement during classes, and allow for flexibility in the length and format of the class. In addition to meeting CM program goals of improving nutrition and cooking skills of participants, Tribal health staff’s desired outcomes focused specifically on supporting participants’ agency to prepare a variety of foods that include both traditional and nontraditional ingredients. They also said that the recipes used in the class should be simple, culturally relevant, and used by participants in the future. Supporting local and tribal food production through the purchase of foods in this program was also mentioned as a desired outcome.

#### Design preliminary adaptations

Cultural relevance was sought primarily through adaptations to the recipes used during each site’s CMAH series. The research team intentionally did not provide a definition of traditional foods to Tribal health staff but rather asked how the staff would define traditional foods and what foods they wanted to see in adapted recipes. Tribal health staff at each site reviewed potential recipes available in the CM Recipe Finder online database (https://cookingmatters.org/recipe-finder/). Each Tribal site was asked to select ≥6 recipes they thought could be adapted to reflect cultural traditions of community members, or that already included foods traditionally eaten in the community. During each lesson one recipe would be tested by the Extension instructor with community participants. Three sites selected recipes in advance, whereas 1 site selected recipes after consulting with participants each week. In total, the 4 sites selected 19 different recipes to use in class and chose 5 to adapt during this preliminary stage. The recipes not chosen for adaptation were included in classes either because they already contained foods that Tribal health staff considered traditional, or Tribal health staff thought they would be popular with participants. Preliminary adapted recipes were formatted into recipe cards for use and testing during each class.

Although the core nutrition lesson content of CMAH curriculum was not adapted in this intervention, each site tailored program implementation methods to best suit its needs. Extension instructors and Tribal health staff coselected lesson topics from the approved CMAH list. Tribal health staff and Extension instructors also worked together to plan class size, timing and frequency of lessons, and recruitment strategies, which varied across sites (Table 1).

**TABLE 1:** Preliminary adaptation implementation methods by Tribal site.

Method	Site 1	Site 2	Site 3	Site 4
Class frequency	Weekly	2 × per week	Weekly	Weekly
Number of classes	6	5	6	5
Unique participants	6	7	6	11
Recipe adaptation method	Preselected recipes that already contained traditional foods	Preselected and then adapted recipes to include more traditional foods	Preselected and then adapted recipes to include more traditional foods	Selected recipes & made adaptations with community participants each week

#### Test preliminary adaptations

The CMAH curriculum and CM recipes were piloted in each of the 4 participating communities. Extension instructors facilitated 4 separate series of cooking and nutrition classes in partnership with staff from the 4 participating Tribes. Between October and December 2022, 3 sites completed either 5 or 6 weekly lessons lasting 1–2 h. The fourth site implemented a condensed series of 5 biweekly lessons. The classes included live instruction, food demonstrations, and opportunities for peer-to-peer learning through facilitated discussions. Each site initially aimed to recruit ∼10 participants to their program, based on space constraints for inperson programming and past experiences with CM participation. Participants were recruited by Tribal health staff through community newsletters, social media, flyers posted at health clinics, and direct outreach to community members participating in other Tribal health programs. At all sites Tribal health staff recruited participants, obtained meeting space, and selected recipes to use during each lesson. Extension instructors facilitated each lesson, purchased groceries, and led cooking demonstrations using the adapted recipes. Tribal health staff engagement varied by site. Staff at 2 sites participated in each lesson and cooking activity. Staff at 2 other sites contributed primarily during the recruitment and planning stages. Table 1 presents key differences in implementation strategies between participating Tribal sites.

#### Refine adaptations

At the end of the final class of each CMAH series, community participants were asked to complete a brief survey of their satisfaction with the CMAH program **(**Supplementary Material 1**)** and to participate in a 1-hour focus group discussion alongside Tribal health staff that same day **(**Supplementary Material 2**).** During focus groups, they were asked to share their perspectives on what foods they consider “traditional,” the cultural relevance of and satisfaction with adapted recipes, their experiences with participating in the CMAH program, and recommendations for improving the program. After participants provided oral consent, all focus groups were recorded via Zoom. Community participants were offered a $25 grocery gift card in appreciation for sharing their time and knowledge during focus groups.

After all series and focus groups had ended, Extension evaluation researchers (ET and DE) interviewed the 4 implementing Extension instructors and 6 of the implementing Tribal health staff, with ≥1 staff interview at each Tribal site. Postintervention process interviews asked about implementation successes and challenges, appropriateness and accessibility of the program and recipes, and recommendations for repeating the program in the future. (Supplementary Material 3).

### Data analysis

Participant retention was tracked and tabulated through weekly attendance sheets. Participant demographics were collected via a preintervention survey and analyzed in Excel. Focus groups and key informant interviews were recorded via Zoom and transcribed. Transcribed data was analyzed following Clarke and Braun’s [21] 6 steps of thematic analysis that included: *1*) familiarization of data, *2*) generation of codes, *3*) combining codes into themes, *4*) reviewing themes, *5*) determining significance of themes, and *6*) reporting of findings. Analysis was completed by 2 independent coders, and themes were generated through consensus. Nutritional analyses were calculated by a nutritional studies student intern for each adapted recipe using data from the USDA National Nutrient Database.

### Ethical review

An ethical review of planned research was conducted through the MSU Institutional Review Board (determined exempt). ITCM obtained project workplan approval from the 4 Tribal governments and shared research materials with Tribes for input prior to implementation. Community participants provided oral consent to take part in focus groups after their respective class series had ended.

## Results

### Community participant characteristics

In total, 30 community participants enrolled in 1 of 4 programs offered, with an average of 7–8 participants per class session. The majority of participants were female, identified as AI/AN, and were aged between 60 and 75 y (Table 2). Eighty-six percent of participants completed every session offered in their series. Nineteen community members participated in postintervention focus groups held in conjunction with their last class.

**TABLE 2:** Community participant characteristics (*n* = 30).

Characteristics	No. participants^1^ (%)
Age (y)	
18–59	12 (40)
60–75	17 (57)
76+	1 (3)
Gender	
Female	20 (67)
Male	4 (13)
Prefer not to respond	6 (20)
Race^2^	
American Indian/Alaska Native	23 (77)
White	6 (20)
Black	1 (3)
Native Hawaiian/Pacific Islander	1 (3)
Prefer not to respond	3 (10)
Reported use of a food assistance program	
Yes	11 (37)
No	19 (63)

1 Includes characteristics of participants from all 4 sites who participated in classes.

2 Participants were able to select more than one race.

### Recipe adaptations

Across the 4 Tribal sites, a total of 8 CM recipes were adapted based on input from Tribal health staff and community participants. Five were adapted by Tribal health staff prior to program implementation. Seven recipes were further refined after focus groups based on community participant input. Adaptations were made to 4 recipes during both the preliminary and refinement stages (Table 3). Eleven additional recipes used in classes were not adapted. Although some recipes were adapted specifically to include more traditional foods, other motivations for making adaptations included local and/or seasonal availability, making ingredients more accessible for people with limited dexterity, and accommodating an individual or group’s taste preferences. Common ingredients selected as traditional substitutes included wild rice, maple syrup, bison, and turkey. Several recipes containing squash were selected without adaptation because they already included traditional food and were locally available during the season of this intervention. Although Tribal health staff from each site selected recipes independently from the other 3 sites during adaptation design, several recipes were selected by multiple sites (Table 3). This suggests some common conceptions across sites of what foods Tribal health staff considered to be traditional.

**TABLE 3:** Recipe adaptations and motivation for adapting.

Original Cooking Matters recipe (n of sites using recipe)	Preliminary adaptation	Adaptation refinement	Adaptation motivation
Roasted butternut squash (3)	--	Use frozen cubed squash instead of fresh to shorten prep time and improve ease of preparation for people with limited dexterity	Accessibility
Fall vegetable salad (3)	--	Make beets optional	Taste
	--	Substitute chard for kale	Availability
	Serve with wild rice	--	Tradition
Turkey chili (2)	Substitute bison or venison for turkey	--	Tradition
	--	Make carrots optional	Taste
Apple crisp (1)	--	Substitute maple syrup for brown sugar	Tradition
Sweet potato shepherd’s pie (1)	Use bison instead of ground beef	Another substitution for beef could be turkey	Tradition and taste
Squash and orzo (1)	Substitute wild rice for orzo	--	Tradition
Bean and vegetable soup (1)	Add corn to make “three sisters” vegetable soup	Substitute hominy for pasta	Tradition
Banana crumble (1)	--	Substitute with maple syrup	Tradition
	--	Add berries and cherries when in season	Availability and Tradition

The following results focus on key themes and recommendations that arose from focus groups and postintervention interviews that occurred during the final “adaptation refinement” stage of program adaptation. Findings are grouped according to how community participants, Tribal health staff, and Extension instructors perceived the adapted recipes’ cultural relevance, implementation of each class series, and their recommendations for future programming.

#### Cultural relevance—community participant perspectives

**FIGURE 2:**
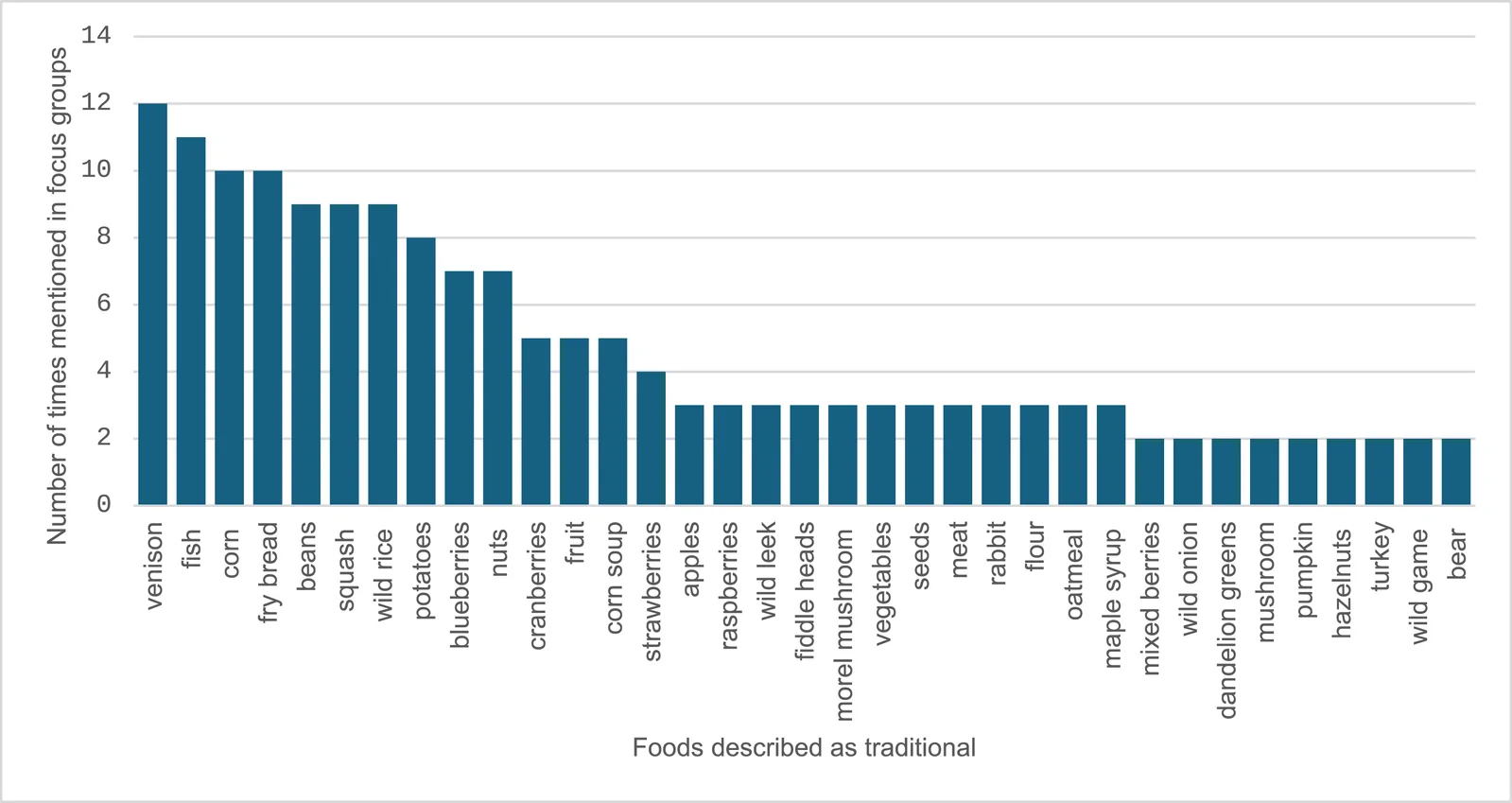
Foods described as traditional by community participants, by number of times mentioned.∗ ∗Other foods that were described as traditional by only one participant included sugar plums, choke cherries, cherries, blackberries, rhubarb, peas, carrots, flint corn, fried potatoes, roots, chaga mushrooms, peppers, cooked spinach, roasted vegetables, beach nuts, pine nuts, acorns, cattails, beef, pork, ham, moose, elk, geese, duck, pheasant, cheese, mac & cheese, chili, barley, wheat, bread, egg bread, white bread, whole wheat bread, squaw bread, Swedish sweet rye bread, flax seed rolls, rolls, corn muffins, pigs-in-a-blanket, Indian Tacos, pie, cakes, banana pudding, sausage dressing, safflower and sunflower oil, buttermilk, gravy, herbs/spices, and honey.

During focus groups, community participants were asked to make a list of the foods that they considered traditional (Figure 2). When asked how they would describe traditional foods, participants shared a variety of perspectives. Some discussed how traditional foods were often passed from generation to generation and found within nature. Recipes appealing to multiple generations were mentioned as well, especially for participants living with kids or grandkids. Sharing food within the community was also identified as a strong tradition. Although there was some overlap in participants’ perceptions of traditional foods, through subsequent discussion, it became apparent that perceptions of cultural relevance and tradition vary greatly between individuals within each community. Participants expressed a wide range of knowledge and behaviors related to food that reflect diverse histories and cultures. For example, some participants were descendants of both Indigenous and non-Indigenous families, such as one community participant who commented,

“You keep using the word traditional, and it makes me think I have to change worlds since I live in two worlds.”

For others, asking a broad question about traditional foods may have offended them, as it implies that their traditions are shared by everyone in the community and reflect a specific perception of Indigenous food culture that may not accurately reflect their own present day cultural preferences. Still, others did not realize that the foods they regularly eat are considered local, traditional foods by others.

When asked about ways to improve cultural relevance of recipes, a community participant suggested that recipes/ingredients should be selected *before* the class starts to gain input from tribal community members to meet the dietary restrictions, food preferences, and preferred cooking methods of the group. Community participants also named a few recipes and/or ingredients to include in future programming: Indian tacos, wild rice, fry bread, corn soup, beans, and pork. Although many participants liked the traditional ingredients used in recipe adaptations, some did not. Ultimately, personal preference most often dictated which foods participants said they prepared and consumed at home. Many participants preferred to eat or try new foods outside of what they consider to be traditional.

#### Cultural relevance—Tribal health staff and Extension instructor perspectives

When asked how to improve the cultural relevance of the program and recipes, one Tribal health staff participating in a focus group discussion suggested inviting a community cultural leader with more indepth knowledge of traditional foods to join or facilitate future classes. Not all the Tribal health staff members participating in this project had the capacity or knowledge to facilitate such conversations. Some Extension instructors and Tribal health staff also agreed with the community participant’s suggestion to choose recipes with participants throughout the duration of the program. The Extension instructor at the one site that did not predetermine recipes said the method was successful.

#### Recipe adaptation challenges—community participant perspectives

Despite general satisfaction with piloted recipes among participants, a common challenge described across sites during focus groups related to accessibility and availability of some recipe ingredients. During focus groups, community participants discussed how traditional foods are expensive and not readily available. As one community participant stated,

“Indigenous foods are often very hard to find and very expensive. There is usually only one or two bags of wild rice at the store, and they’re usually $10 a piece.”

Additionally, community participants shared that some foods considered traditional by community members may not be available because they are not typically part of the conventional United States or local food system. As one focus group participant shared,

“If you want to eat traditional foods, you really gotta have connections to find this stuff. Because the majority of people are not going to go harvest it or hunt it themselves. And a lot of it you can’t buy in the store.”

#### Recipe adaptation challenges—Tribal health staff and Extension instructor perspectives

During postintervention interviews, Extension instructors, as well as Tribal health staff, described access to traditional foods as a barrier to implementing some recipe adaptations. They shared that sometimes, traditional food was not reasonably accessible (either physically or economically) for participants or instructors to use during a class.

#### Program implementation—community participant perspectives

Among the 19 community participants who joined focus groups and completed a program satisfaction survey, most expressed positive experiences with the program. All said they were “very satisfied” with program registration, interaction with instructors, and recipe preparation activities. Most were “very satisfied” with program length (16 participants) and content of lessons (17 participants). Two were “somewhat satisfied” with program length and content. One person said they were not at all satisfied with the length of the program, although they did not indicate if they thought the class was too long or too short. All focus group community participants said they were “very likely” to recommend the program to family and friends.

During focus groups, community participants stressed the value of ensuring continuity in Extension program staffing and the importance of building and maintaining trust with Extension program instructors. They also identified a preference for continual, consistent, hands-on, interactive programs. These could be programs that are ongoing with a variety of topics and not limited to 5 or 6 wk.

Relationships formed between participants were also seen as a benefit to participating in the program. Participants enjoyed being able to develop relationships with other community members in the group and learning from each other’s experiences. When asked what they enjoyed most about the program, community participants at one site responded, “interacting with everybody,” “being able to be together,” and “just cooking together as a group.”

Offering a program that was not sequential in nature also allowed participants to miss a week if needed without falling behind. One focus group community participant corroborated this impression by saying,

“You didn’t have to be here last week to know what’s going on this week. You know, you didn’t get lost…If somebody wanted to walk in and sit in on the class, they could learn [how to cook] a meal.”

#### Program implementation—Tribal health staff and Extension instructor perspectives

During postintervention interviews, some Tribal health staff observed that although the classes were smaller than they had intended, that may have been a reason for the high participant retention and satisfaction. Smaller class sizes of 6 or 7 instead of the expected 10–15 at 3 of the sites allowed for more flexibility in the location of programs as well as potentially building stronger relationships between community participants. One tribal partner suggested that smaller classes may have helped community members feel more comfortable attending each week, considering that the program started soon after the official end of the COVID-19 pandemic, when some people were still wary of attending social events in the community.

Tribal health staff and Extension instructors shared additional impressions of why the classes were successful. Reasons included Extension instructor productivity and flexibility, community participant comfort with the Extension instructor, communication between the Extension instructor and the Tribal health staff, and texting or emailing class participants every week to communicate reminders about class. When discussing ways to improve participant engagement with future programs, one Tribal health staff partner suggested,

“To keep offering [classes] and be more lenient on the number that attend. It takes at least two years to build up any consistent programming to get good numbers…It takes a while to build up the momentum.”

This sentiment echoed the similar comments made by community participants in focus groups, as previously described.

Other opportunities to improve the class model in the future suggested by Tribal health staff and Extension instructors included reducing paperwork burden further, providing recipes and handouts printed in color, having a centrally located and well-stocked kitchen for programs, planning sufficient time for word-of-mouth participant recruitment, providing local food assistance program resource information to participants, and teaching other nutrition concepts of interest to the group.

## Discussion

Each site experienced its own unique challenges and successes when piloting the program and adapting recipes. A key challenge experienced across sites was that local traditional foods may be hard to find and costly for participants, which poses a barrier to including them in recipes and the program. This finding is consistent with Brown et al. [18] findings from an adaptation in the same geographic region. They suggested that adaptations address the contextual factors that affect dietary behaviors, like food access. In a review of 66 nutrition interventions adapted for cultural appropriateness in Indigenous communities, Vincze et al. [22] noted that more interventions focused on creating a healthy food environment are needed, recognizing the limitations of nutrition education alone. Combining these educational programs with other policy, systems, and environmental approaches to improving Indigenous food access and food sovereignty may help to address traditional food access challenges. Although the adapted recipes developed through this project may be useful references for others who wish to do this in the same or similar communities, this is not a one-size-fits-all approach to implementing nutrition and cooking education programs in tribal communities. Although many other interventions have primarily used visual, surface level adaptations to intervention content, this research specifically aimed to adapt the cooking elements of the class by seeking input from community members and basing recipe adaptations and refinements on their experiences and values [22]. It is important that future programs make space for both the shared experiences and cultural traditions of each community, as well as the individual traditions and preferences of participants. Program facilitators should be intentional about balancing a desire to support local traditional foods in the broader community with meeting the needs and preferences of individuals. For Extension instructors, getting to know the community members, approaching activities and participants with curiosity rather than sharing “expert” advice, and seeking ways to do more continuous programming may help to achieve this balance and best meet community needs.

### Strengths and limitations

To our knowledge, this is one of the first documented efforts to adapt national evidence-based SNAP-Ed-approved curriculum to improve cultural sensitivity to Anishinaabe communities in Michigan. Using and adapting an existing SNAP-Ed-approved curriculum allowed for faster implementation than developing a program from scratch. Due to the positive experience that staff and community members had with the program and new partner relationships built between MSU Extension and Tribes, additional collaborative SNAP-Ed-funded programs had been implemented at 3 of the 4 sites as of the writing of this article. Two sites continued to implement CM curricula. The adapted recipes have been used in similar SNAP-Ed-funded programs with other Tribes in the state since completion of the initial project.

MSU Extension is an 1862 Land Grant Institution staffed primarily by non-Indigenous educators. The research team responsible for planning and implementing evaluation of this project was led by non-Indigenous researchers who are trained in Western scientific methods of research. Therefore, this work is based on a scientific framework for intervention adaptation design used in an Indigenous context rather than based on Indigenous methods and ways of knowing. As partners, Tribal health staff were consulted during formative research and throughout the project, from recommending initial recipe and process adaptations to providing review and feedback on results and communication of outcomes. Tribal health staff were familiar with the communities they served, but they were not necessarily all members of the participating Tribes themselves, so they may have had different understandings of what is considered culturally relevant. It is possible that involving community members as well as Tribal health staff from the project’s conception may have yielded more diverse results or a richer understanding of how different community members view traditional foods. Finally, because 19 out of 30 program participants joined the postintervention focus groups, some key community perspectives may have been left out during evaluation. However, similar themes emerged across the 4 focus group sites, suggesting some saturation in responses. Because of a small sample size and the qualitative nature of this research, findings are intended to be considered as examples rather than suggest a one-size-fits-all approach to adapting nutrition education programs in Indigenous communities.

### Lessons learned and recommendations for further refinement

The high retention rate (86%) was a positive and unexpected outcome of this program. There can be many barriers to adult participation in educational programs, such as family obligations and lack of time, particularly for low-income audiences such as those who participate in SNAP-Ed [23]. Although the classes were relatively small in this program, most participants who started the class consistently came back each week, therefore receiving the most potential benefit from the class series. The flexibility of Extension instructors, as well as Tribal health staff partners, and willingness to adapt the program format and logistics to meet communities where they are were positive findings overall.

Adapting a program to be culturally appropriate to people whose traditional cultures have been systematically diminished over generations poses unique challenges. The systematic erosion of traditional American Indian cultures through policies of removal and boarding schools cannot be separated from education and cultural discussion in the present day [24]. This program evaluation highlighted how program adaptation can be a way not only to improve engagement and retention but also to strengthen and revitalize cultural elements that may have been lost or diminished within a community. For example, in the 2009–2013 American Community Survey, 11,685 people said they speak either Ojibwa, Potawatomi, or Ottawa at home [25]. Very few speak one of those languages as their first language. Thus, incorporating Anishinaabemowin words in program materials and recipes could be a way to reintroduce the language to community members and support those who do speak the language rather than adapting for the purpose of assuring comprehension of educational materials.

The historical erosion of traditional Anishinaabe food systems also presents logistical challenges for adaptation. The expense or unavailability of some traditional ingredients were significant barriers to piloting and community member uptake of adapted recipes. Many of the grocery stores located in or near participating communities either did not carry desired traditional foods or had limited stock at high prices. To address this challenge, it may be helpful to partner with local farmers, community gardens, or agricultural Extension educators to improve access to these foods. Educational programming could also be paired with other initiatives focused on improving access to healthy, traditional foods. Expanding efforts to include more traditional foods in food distribution programs could also support the availability and access of such foods for community members with the most need [26]. The FDPIR Self-Determination Demonstration Project, for example, provides more flexibility for tribal food distribution sites and enables them to purchase and provide more tribally produced foods to community members [26]. For foods that are not typically available in stores or food distribution sites because they are harvested, foraged, or hunted, “bundling” educational programs that improve knowledge and access to traditional harvesting and storing methods alongside or in addition to nutrition and cooking classes could be beneficial. Adapting a nutrition education and cooking class to include traditional foods poses an opportunity more broadly to align nutrition education programs with local tribal food sovereignty initiatives that aim to restore access to and control over traditional foods in Tribal communities.

We observed considerable intra-Tribal diversity in participants’ history, traditions, foodways, cultural norms, and level of access to traditional and healthy food across all sites. The culturally sensitive approach taken in this intervention allowed us to acknowledge historical context while also making room for individual histories and preferences. Historical levels of assimilation into colonizer food culture vary within communities, resulting in differing familiarity with and interpretation of traditional foods and cultural practices [27].

Focus groups with community members also made it clear that traditional and cultural are not necessarily the same, which aligns with Rocillo-Aquino’s definition of traditional food encompassing place, time, know-how, and cultural meaning [2]. Consequently, adapting to be relevant and sensitive to a contemporary culture may look different than exclusively adapting to highlight and revitalize traditions. For example, fry bread was one of the most commonly listed traditional foods by focus group participants, but there is debate across Indian Country about whether it should be considered “traditional” because its origins are rooted in colonization and commodity foods rather than foods Indigenous to American Indian communities [27]. Nevertheless, some might consider it a cultural food because it is often eaten at celebrations and community gatherings. This project demonstrated the complexity of cultural adaptations, the need for cultural sensitivity, and program facilitators approaching as curious learners rather than experts.

Future iterations of this and similar programs must account for the difference between, and make space for, both cultural and traditional preferences and needs of communities. Given the diversity of histories, cultures, and knowledge within Tribal communities, meeting the needs of contemporary Tribal community food culture should be balanced with efforts to revive traditional foodways and knowledge. To understand and more fully include local culture*,* there should be more engagement with community members before an intervention begins, when selecting recipes and adaptations. More incorporation of and understanding of tradition could mean inviting a knowledge keeper or cultural leader from the community to share with the class and provide a perspective and knowledge that not all community members and Tribal health staff may have. Cofacilitating with a community expert is not uncommon in CM programs because the program model originally paired a nutrition educator with a local chef who is more knowledgeable and skilled in the cooking portion of the program. Involving a variety of community perspectives that include input from cultural knowledge keepers has been shown to be an effective way to design culturally sensitive health interventions with Indigenous communities [15,17]. Finally, for a deeper understanding of local community needs and cultural norms, programs should be consistent, long-term, and interactive, supporting strong relationships with community members rather than a time-limited, in-and-out approach to programming. Sustained, authentic relationships with Tribal communities are fundamental for continued trust and to address health disparities through collaborative and culturally sensitive programs.

## Author contributions

The authors' responsibilities were as follows – ET, DE, and RJG: designed evaluation research, conducted research, and analyzed data; and ET, DE, RJG, and BS: wrote the paper; ET: had primary responsibility for final content; and all authors: read and approved the final manuscript.

## Funding

The intervention was delivered by Michigan State University (MSU) Extension staff funded by the United States Department of Agriculture’s (USDA) Supplemental Nutrition Assistance Program (SNAP) and the Michigan Department of Health and Human Services. MSU Extension staff worked collaboratively with tribal partners and the Inter-Tribal Council of Michigan, with funds from the Walmart Healthy Native Foods grant being used for focus groups and participant groceries. Funders played no role in study design, data collection, analysis, or interpretation of data.

## Data sharing

Data described in the manuscript, code book, and analytic code will be made available upon request, pending approval of each participating Tribal organization.

## Conflict of interest

The authors report no conflicts of interest.
