# Supplementary material for: Lessons Learned from Implementing Culturally Sensitive Adaptations to a Nutrition Education Program in Four Anishinaabe Communities
Source: Curr Dev Nutr. 2024 Oct 29;10(Suppl 2):104496. doi: 10.1016/j.cdnut.2024.104496 (PMC13615722; doi:10.1016/j.cdnut.2024.104496)
Supplement: Supplementary file 1 [file mmc1.docx]

**SUPPLEMENTARY MATERIALS**

**Supplementary Material 1: Participant Program Satisfaction Survey**

1. Mark how satisfied you were with the following parts of this program (select not at all satisfied, somewhat satisfied, or very satisfied for each part)

|  | Not at all satisfied | Somewhat Satisfied | Very Satisfied |
| --- | --- | --- | --- |
| Registration |  |  |  |
| Length of the Program |  |  |  |
| Interaction with instructors |  |  |  |
| Content & recipes |  |  |  |
| Recipe preparation activities |  |  |  |

2. How likely are you to recommend this program to a friend or family member? (not at all likely, somewhat likely, very likely)

|  | Not at all likely | Somewhat likely | Very likely |
| --- | --- | --- | --- |
| Likely to recommend this program to a friend or a family member |  |  |  |

**Supplementary Material 2: Participant Focus Group Questions**

**Opening question**

1. Introduce yourself and share a family food memory/recipe

**Traditional Foods/Practices**

*Ask them to use the pen and paper in front of them for this activity.*

2. On your own, write down some words that you would use to describe “traditional foods.” It doesn’t have to be in complete sentences.
3. Now, looking at what you wrote down, list as many foods as you can that you would consider traditional.
4. Circle the foods that you have access to in your community.

*As a group, ask a few people to share what they came up with.*

5. Prompt: What words did you use to describe “traditional”? What foods did you include?

**Cultural Acceptability – Curriculum**

Thinking about the content you learned in the last 6-weeks from the Cooking Matters at Home program:

6. How did the program cover topics that are relevant to your daily life?
7. What, if anything, did you learn about traditional foods?
8. How would you change the content (lessons, visuals) to better fit with your cultural food practices?

**Cultural Acceptability - Recipes**

*Provide the six recipes to participants and ask them to review them on their own to recall what they did.*

9. Which recipe would you be most likely to use at home? (Why?)
10. Which one would you be least likely to use? (Why?)

*Point out each recipe and ask:*

11. How likely are you to use the ‘traditional’ ingredients from these recipes?
    a. What barriers would prevent you from using them?
    b. What would you substitute them with that might be more accessible to you?
12. What other changes would you make to the recipe based on the foods you normally eat? Consider:
    a. Ingredients
    b. Cooking methods
    c. Equipment

**Closing**

13. What did you enjoy most about this program?
14. What did you enjoy the least?
15. What else would you like to share about your experience with this program and/or the recipes?

**Supplementary Material 3: Process Evaluation In-Depth Interview Questions**

**Introduction**

1. Can you tell me briefly about your current role in your organization?
   a. How long have you worked in that role?
2. What role did you play in implementing this Cooking Matters program?
3. Have you implemented or supported a Cooking Matters program in the past? (If so, how was this experience different?)
4. Overall, how would you say the program went?

**Participation**

When we spoke before this program began, you said that you would like to see strong participation in this program. I’d like to ask a few questions to follow up on that.

5. How would you describe the recruitment process for this program?
   a. *Prompt:* How did you encourage people to show up*?*
6. How would you describe the experience of recruiting participants?
   a. *Prompt:* What challenges/successes did you encounter? Why?
7. What would you do differently to recruit participants if you could do this again?
8. ____ people who signed up attended all or most of the sessions. What do you think helped them to return each week?
9. What would you do differently to improve retention if you did this again?

**Implementation**

Now let’s talk about the implementation of this program. (By implementation, I mean the process of preparing lessons, purchasing groceries, and co-facilitating the lessons and cooking activities each week).

10. What went well during implementation?
    a. Why did it work well?
11. Please describe any challenges you encountered during implementation.
    a. Why did those challenges occur?
    b. How did you address the challenges?
12. What would you do differently next time?

**Appropriateness**

As you know, a goal of this project was to adapt it to be culturally relevant and accessible to the tribal community you serve.

13. Overall, how much do you think the program as implemented aligns with the community’s cultural beliefs and traditions?
    a. *Prompt:* What aspects of the program seemed to conflict with or contradict your community’s cultural norms and traditions?
14. What, if anything, do you think could make this more culturally appropriate for the community?

**Capacity/Sustainability**

I’d like to understand more about your capacity to do something like this in the future.

15. Which aspects of this project required the most effort or resources from you?
    a. Were you surprised by this?
16. How likely is it that you will do this program again with the resources you currently have?
17. What additional resources (time, staff, money, knowledge) would you need to do this program again/better?
18. How would you describe the partnership between MSU Extension and tribal staff for this project?
19. What could MSU Extension do to sustain this partnership?

**Effectiveness**

20. How will this program help your community members improve their nutrition and physical activity behaviors?
21. What barriers do you think your community members might encounter in using what they learned in this program to make behavior change?

**Closing**

Thank you for taking the time to talk with me, and for your participation in this program.

22. What else would you like us to know about this program?
